# In elderly individuals, the effectiveness of sensorimotor training on postural control and muscular strength is comparable to resistance-endurance training

**DOI:** 10.3389/fphys.2024.1386537

**Published:** 2024-08-21

**Authors:** Mikuláš Varjan, Ľubica Žiška Böhmerová, Ľudmila Oreská, Peter Schickhofer, Dušan Hamar

**Affiliations:** ^1^ Department of Biological and Medical Sciences, Faculty of Physical Education and Sports, Comenius University in Bratislava, Bratislava, Slovakia; ^2^ Centre of Active Ageing, Faculty of Physical Education and Sports, Comenius University in Bratislava, Bratislava, Slovakia

**Keywords:** aging, seniors, balance abilities, strength abilities, physical performance

## Abstract

While classical resistance exercise is an effective way to improve strength and control postural sway, it may not be suitable for some elderly individuals with specific health disorders (e.g., aneurysms). Therefore, there is a need to explore alternative modalities. The study aimed to evaluate the effects of sensorimotor training on muscle strength and postural control in the female elderly population and subsequently compare these effects with a traditional combined resistance-endurance training program. A total of 34 healthy, active elderly women aged from 65 to 75 years, (average age 72.7 ± 4.4 years, height 161.6 ± 5.1 cm, and weight 66.9 ± 8.4 kg) were randomly assigned to three groups undergoing different 10-week interventions: the resistance-endurance training (RET, n = 11), the sensorimotor training (SMT, n = 12) and the control group (COG, n = 11). Prior to and after the interventions all participants underwent tests of maximal voluntary contraction of the dominant and non-dominant leg; postural sway tests with open and closed eyes; novel visual feedback balance test; 10-meter maximal walking speed (10 mMWS) and stair climb test. A T-test and repeated measures ANOVA were used, followed by the Bonferroni *post hoc* test, to compare the pre and post-measurements and assess differences in gains between groups. Results showed a significant main effect of time on strength (*p* < 0.001). In addition, significant differences in time × group interaction on strength (*p* < 0.01), postural control (*p* < 01), and ascendant and descended vertical speed (*p* < 0.001) were observed. Besides, the RET group improved significantly the maximal voluntary contraction of both dominant (16.3%, *p* ≤ 0.01) and non-dominant leg (10.9%, *p* ≤ 0.05). SMT group improved maximal voluntary contraction of both dominant (16.6%, *p* ≤ 0.001) and non-dominant leg (12.7%, *p* ≤ 0.01). In addition, they also improved mean velocity of the centre of pressure (COP) in postural sway test with eyes open (24.2%, *p* ≤ 0.05) as well as eyes closed (29.2%, *p* ≤ 0.05), mean distance of COP in novel visual feedback balance test (37.5%, *p* ≤ 0.001), ascendant and descended vertical velocity (13.6%, *p* ≤ 0.001 and 17.8%, *p* ≤ 0.001, respectively). Results show not only resistance training but sensorimotor intervention boosts strength too. This intervention also enhances postural control and functional abilities for both ascending and descending movements.

## 1 Introduction

Around 11% of the world’s population is currently aged 60 or older, and this is expected to double to 22% by 2050 (Kanasi et al., 2016). Thus, there is a need to find better ways to support healthy ageing (Witham et al., 2022). Physical activities, especially resistance training, are known to promote healthy ageing. Recent studies suggest that combining resistance training with endurance activities is even more beneficial. However, some people may find this type of exercise unsuitable. As a result, the scientific community is actively exploring alternative exercise options that are more efficient and effective for promoting healthier ageing. One such promising option is sensorimotor training.

It is important to realise, that as people age, their physical abilities naturally decline, leading to various challenges and limitations in their daily lives (Maresova et al., 2019). Physical fitness has a significant impact on life expectancy, as it determines a person’s capability to independently carry out daily activities without difficulty or assistance (Gill et al., 2016). One notable consequence of aging is the loss of muscle mass, known as sarcopenia, which directly corresponds to a decrease in muscle strength, referred to as dynapenia (Tanaka et al., 2017). Yet, several factors contribute to the inevitable progression of sarcopenia, including dysfunction in mitochondrial and autophagy processes, as well as the limited ability of satellite cells to regenerate. The natural decline in muscle mass and motoneuron function that occurs with ageing (Agostini et al., 2023). Both sarcopenia and dynapenia contribute to a decrease in overall physical fitness and changes in general health status (Garatachea et al., 2015; Tieland et al., 2017). Starting at the age of 60, the ability to generate force through isometric contractions decreases by approximately 1.5% each year (Jubrias et al., 1997). However, both sarcopenia and dynapenia have a more significant impact on a muscle’s ability to generate force during movement (Clark and Carson, 2021). Conversely, extensive research supports the notion that regular exercise results in skeletal muscle hypertrophy, increased muscle strength, and enhanced overall physical performance and fitness (Bekfani et al., 2016; Mcleod et al., 2024). Additionally, when planning regular physical activity, it's important to also maintain a balanced diet, which is rich in antioxidants, polyunsaturated fatty acids, vitamins, probiotics, prebiotics, proteins, and short-chain fatty acids. Specifically, paying attention to the intake of vitamin D is crucial as low levels of this vitamin are linked to an increased risk of sarcopenia (Remelli et al., 2019; Agostini et al., 2023; Widajanti et al., 2024). In addition, studies by Agostini et al. (2023) highlight that vitamin D supplementation is particularly beneficial in stimulating skeletal muscle hypertrophy and promoting protein synthesis. Therefore, it is highly important to combine a well-balanced diet with vitamin D supplementation, alongside regular physical activity, to effectively protect aging muscles from the development of sarcopenia.

Besides experiencing a decline in physical strength, the elderly also tend to face a deterioration in their balance skills (Clemson et al., 2012), leading to a significant decrease in their level of physical activity. All these declines are slowly leading to a potential fear of falling and the subsequent risk of injury. Thus, it is important to note that maintaining balance relies on the integration of sensory information from multiple systems, including the somatosensory, visual, and vestibular systems, as well as the neuromuscular system (Hansson et al., 2010; Peterka, 2018). This system incorporates neuroregulatory mechanisms like proprioception and intramuscular coordination, which play a significant role in boosting force production and overall performance. These mechanisms enable the engagement of a greater number of motor units and the efficient utilization of the produced force to complete tasks (Marshall et al., 2022). As a result, as we age, it becomes crucial not to overlook the importance of balance for overall physical performance. By improving their balance capabilities, older individuals can greatly enhance their ability to carry out daily activities, as well as increase their potential for generating force (Willardson, 2007). There are various training options available to enhance the mechanisms mentioned above. Classical resistance training, for instance, remains a key component for improving strength (Kraemer and Ratamess, 2004; Hortobágyi et al., 2021). Another frequently utilized option is aerobic training, which primarily focuses on cardiovascular fitness. However, it is worth noting that these forms of training may not be suitable for every elderly individual. Consequently, sensorimotor training that is physically less demanding should be considered as an alternative approach for improving balance and potentially enhancing strength capabilities.

Sensorimotor training is a specialized type of training that aims to improve coordination and movement control by integrating the sensory and motor systems. Certain studies suggest that sensorimotor training could be beneficial in improving postural sway, balance ability, and coordination in healthy individuals (Bruhn et al., 2004; Di Corrado et al., 2023). However, there is limited research on its effectiveness for senior citizens. Although it can enhance motor skills, it is still uncertain whether sensorimotor training directly increases muscle strength. Thus, additional research is necessary to determine the specific impact of sensorimotor training on strength.

The main objective of the study was to investigate the effects of sensorimotor training on muscle strength and postural sway control in elderly women and subsequently compare these effects with a traditional combined resistance-endurance training program.

## 2 Methods

### 2.1 Subjects

A total of 34 female older adults aged from 65 to 75 years (average age 72.7 ± 4.4 years, height 161.6 ± 5.1 cm, weight 66.9 ± 8.4 kg) with no previous experience with exercise, participated in this study All participants were supposed to have no history of regular physical activity training and no more practice than 150 min of moderate or 75 min of vigorous intensity per week. They also were not supposed to perform any regular active recreational sports exercise for at least 3 years before attending the study. All female participants were randomly assigned to the following three groups: 1. group that took part in a 10-week resistance-endurance program (RET), 2. group that underwent sensorimotor training (SMT), and 3. control group (COG) which did not undergo any intervention. Prior to the study inclusion criteria, all female participants were healthy and free from severe mobility impairments and any cardiovascular, neurological, or other chronic diseases that could interfere with any of the training programs. Before pre- and post-intervention testing, participants were strictly verbally and writenely informed by the research supervisors not to take any medication, such as pain drugs, stimulants, etc., or caffeine during the testing day, as it could falsely affect the functional status during muscle strength and physical performance assessments. On the testing day, all participants had to declare verbally their actual health status. All participants provided written informed consent before the beginning of the study and were notified of their rights to withdraw from the study at any time. The study protocol followed the ethical guidelines of the Declaration of Helsinki 2000, and its later amendments from 2013. The study was approved by the ethics committee of the Faculty of Physical Education and Sport, Comenius University in Bratislava (4/2023).

The exclusion criteria were the following:

• Present disorders, injuries, or impaired mobility related to the musculoskeletal system.
• Present acute or chronic infections that would prevent individuals from participating in the study.
• Present cardiovascular, neurological, cancerous, metabolic, autoimmune, or other diseases that would hinder an individual´s participation in the study.
• Individuals with confirmed or suspected malnutrition.
• Significantly underweight Individuals.
• Individuals receiving artificial administration of hormones, for example, insulin or drugs to support immunity.

### 2.2 Procedures (study design)

**FIGURE 1:**
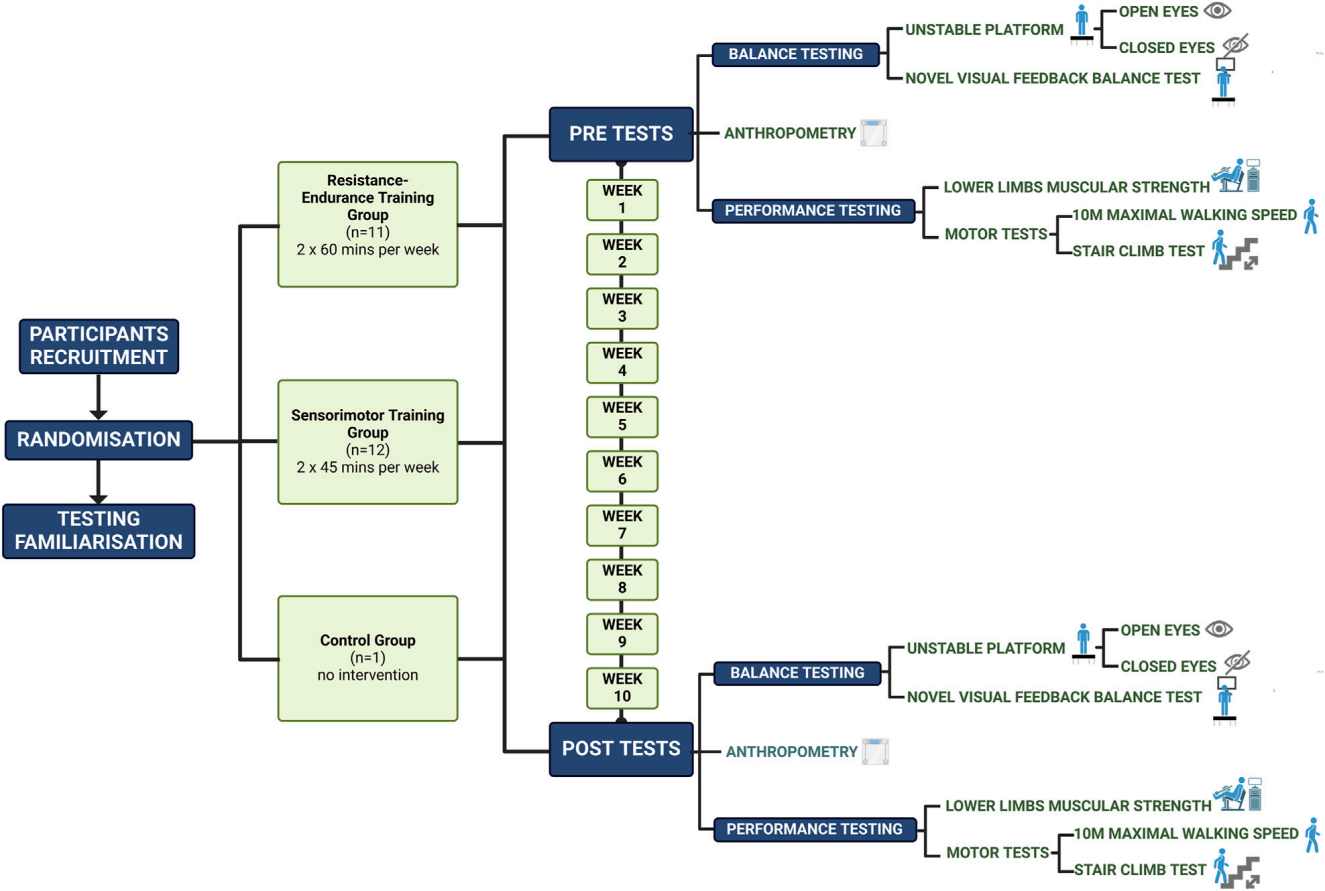
Study design (Created with BioRender.com).

Both experimental training programs were conducted by experienced coaches who have previously worked with older adults and were carried out at the Centre of Active Ageing (CAA). Prior to the study, all female participants attended a session to be familiarized with the testing procedures by the study researchers. A week before the training programs began, the participants underwent body composition and physical fitness assessments, including measurements of muscle strength in their lower extremities, motor skills, balance, and coordination abilities. The post-test measurements were taken within a week after the training period concluded. Both testing sessions were performed at similar time points (between 08:00 a.m. and 10:00 a.m.), and the order of tests was strictly set. All tests were conducted at the CAA under the supervision of two or three researchers with previous testing experience. The whole study design is illustrated in Figure 1.

### 2.3 Resistance - endurance training

The intervention aims to improve the movement system, movement literacy, and overall fitness of seniors by integrating new levels of physical activity into their daily routine. The program is based on the principle of “movement-based training” and includes a combination of complex pulling and pushing exercises for the upper and lower body. The training program mainly included exercises such as free weight sit-to-stand (or using a kettlebell), kettlebell deadlift, leg press, leg extension (lower body exercises), and their various modifications (progression or regression). The upper body exercises consisted of box push-ups, horizontal chest press, TRX Row, horizontal Keiser bilateral Row, and their various modifications (progression or regression). All the individual exercises are part of the Centre of Active-Ageing training manual. Each set (3) consists of 10–12 repetitions using a load equivalent to 10–12 repetition maximum (RM). The first session was used to establish individual baseline training loads. Progressive overload was utilized on a bi-weekly basis by adding 2%–5% of estimated 1RM, based on the individual’s ability to perform the current workload. The endurance component of the program is based on “conditioning - interval training” (Laursen and Buchheit, 2019) using equipment such as stationary bikes, airbikes, and exercises like stepping, running, and sled pushing in 6 sets with a load-rest interval ratio of 1:2. All training sessions include a 10-minute warm-up and a 5-minute relaxation after exercise.

### 2.4 Sensorimotor training

**FIGURE 2:**
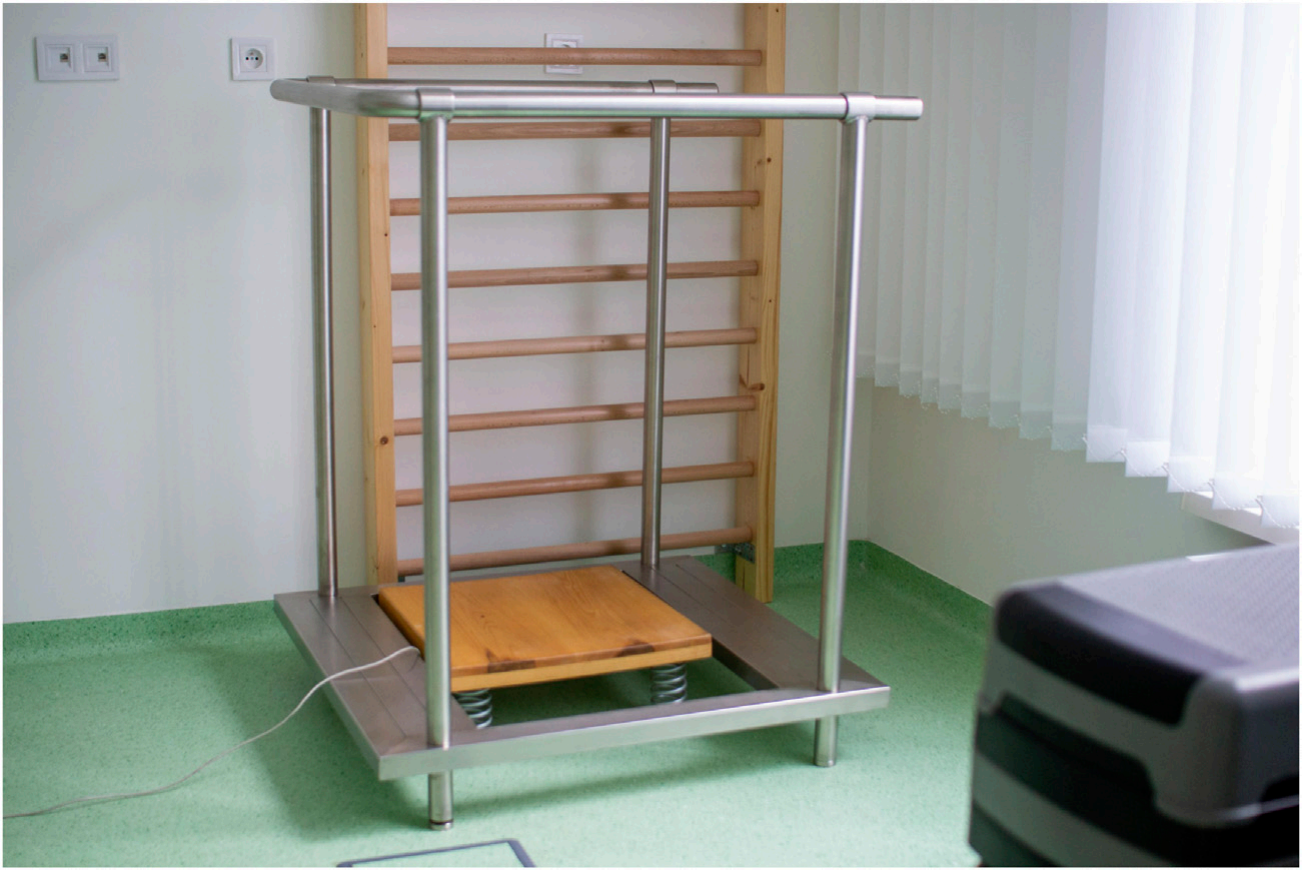
Unstable stabilographic platform.

Sensorimotor exercises (Hamar 2008) were performed on an unstable spring supported stabilographic plate (Figure 2) (Fitro Angle Sway Fitronic, Bratislava, Slovakia). The system monitors projection of body’s center of gravity (COG) horizontal movements to the ground referred to as center of pressure (COP) and displays it online on the screen (Hamar, 2018).

Three routines were applied using this system.

**FIGURE 3:**
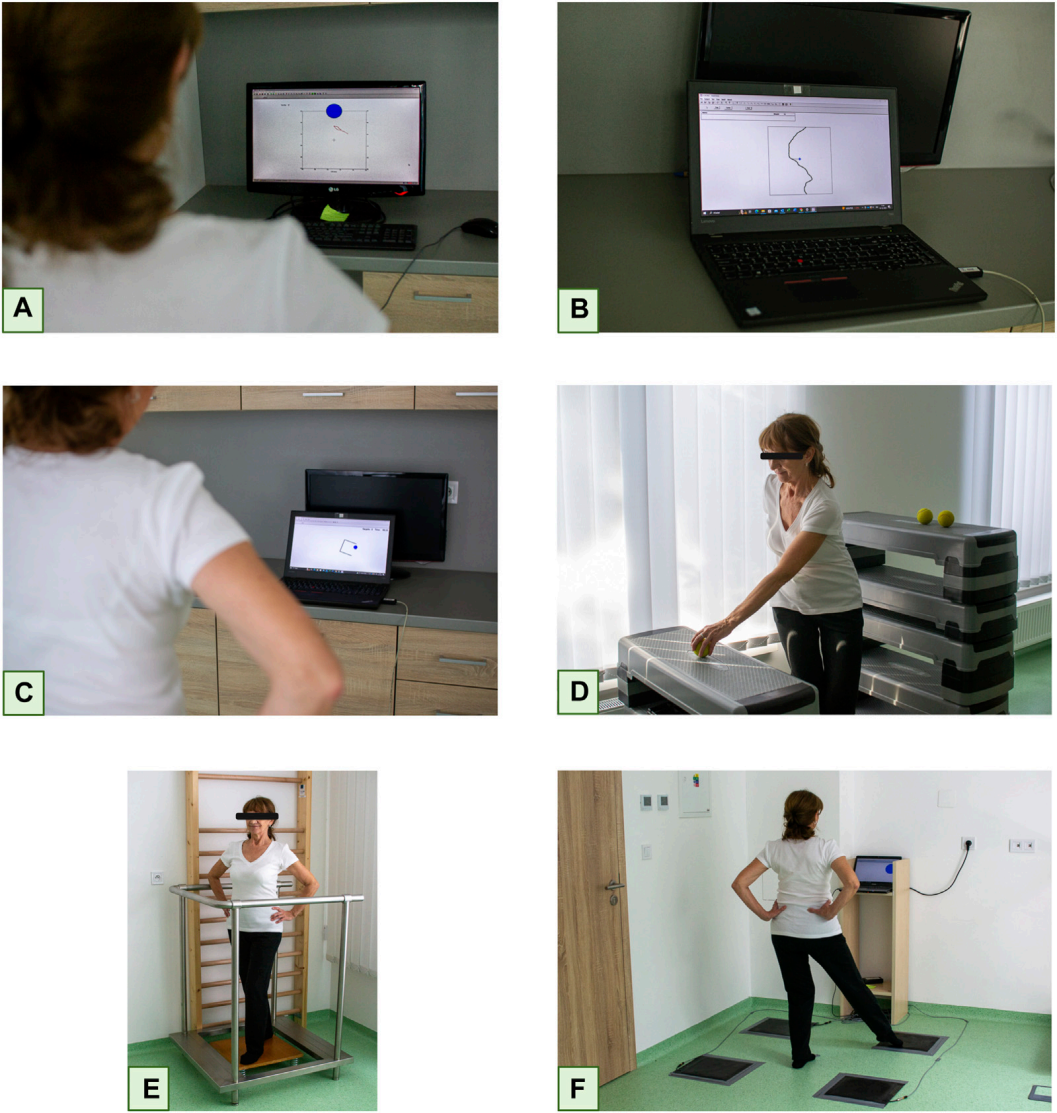
Sensorimotor Training Session Legend: Following the curve **(A)**, Hitting randomly generated targets **(B)**, Shifting a small circle into the “cup” **(C)**, Still stance on an unstable platform without visual control **(D)**, Replacing an object from a higher pad to a lower one in different stance positions **(E)**, Stepping as fast as possible on the appropriate contact plate **(F)** (Created with BioRender.com).

In the first one, the individual has to, by moving their own body in a horizontal plane, follow the curve generated by the computer and displayed on the monitor screen (Figure 3A) as closely as possible.

In the next exercise, randomly generated targets have to hit with online displayed COP while moving body in horizontal position (Figure 3B).

In the third exercise, a small circle, representing the COP has to be guided into the “cups” generated on the screen, again by shifting body in horizontal plane (Figure 3C).

The values achieved in each of the routines after their completion were displayed to motivate subjects. They were average distances of the COP from the moving curve (first routine), the total path, and the time required to hit the target with the virtual COP in the second and moving virtual COP into the “cup” in the third routine.

In addition to these three routines, participants perform two additional exercises on an unstable plate without visual feedback. In the first exercise, the individuals performed different positions without visual control (Figure 3D). In the second exercise, they were again tasked with transferring an object from a higher pad to a lower one in different positions (Figure 3E). In the last exercise, the participants had to step as quickly as possible on the appropriate contact plate (Fitro Agility Fitronic, Bratislava, Slovakia) in response to a stimulus displayed in different corners of the computer monitor (Figure 3F). The average reaction time was calculated from the total number of reactions. The whole session was based on the principle of circuit training in three series with 30 s of load and 30 s of rest. Every second week, the individual exercises were made more difficult by one level according to the protocol report, and the duration of load was increased by 5 s.

### 2.5 Test protocols

#### 2.5.1 Body composition

Body height was measured using a digital free-standing stadiometer InBody BSM 170 (InBody Co., Ltd., Cerritos, CA, United States) and determined to the nearest centimetre Participants’ body weight, muscle mass (kg), and fat mass (kg) were analysed using the bioimpedance method on InBody 230 (InBody CO., Ltd., Cerritos, CA, United States). The body weight was set with participants wearing underwear. Afterwards, the BMI was calculated.

##### 2.5.1.1 Balance test on unstable platform

An unstable stabilographic platform (Fitro AngleSway Fitronic in Bratislava, Slovakia) was used to assess the stability of posture by analyzing the Centre of Pressure (COP) (Ruhe et al., 2010). The unstable posturographic system based on the platform, supported by 4 springs (coefficient of elasticity f - 10 N/mm) and equipped with biaxial inclinometric sensor features comparable reliability r = 0.932 (eyes open), and r = 0.868 (eyes closed) (Hamar, 2018), with traditional stable and force transducer based posturographic system (r = 0.920 and r = 0.887 respectively) (Zemková and Hamar, 1998), hence represents a reliable and valid alternative for the assessment of balance capabilities (Zemkova and Hamar, 2015). The main differences to traditional systems involved using inclination sensors instead of force transducers to calculate instant COP (Hamar, 2018). To evaluate the postural sway, the velocity mean (VM) of the COP was used. During the test performance, participants were instructed to stand barefeet on the unstable stabilographic platform with their feet hip-width apart and toes slightly pointing outwards. Throughout the 30-second trial, participants were instructed to keep their hands on their hips and knees extended and to minimize their body movement as much as possible. The participants performed a total of three 30-second trials, with a one-minute rest interval between trials. The result was determined based on the best trial’s outcome. The test was carried out using two distinct ways - one with visual feedback and another without it (Sedliak et al., 2013).

##### 2.5.1.2 Novel visual feedback balance test

Participants stood on a computer-based stabilographic unstable platform (Fitronic, Bratislava, Slovakia) in a parallel stance with their bare feet shoulder-width apart, toes slightly pointing outwards, and hands on the hips. A computer screen was positioned about 1.5 m from the platform at the subject’s eye level. Instantaneous visual feedback of participant’s COP was given in the form of a blue cross visible on the screen. Participants were asked to keep the blue cross’s movement as close as possible to a predefined flowing curve by moving their bodies the curve was moving from the top of the screen downwards, and the subjects copied its shape by their body movements (COP displacement) in the mediolateral direction (ML). The curve parameters were programmed with custom-made software and were identical in all tests (Hamar 2008). The system monitored the horizontal distance (mean distance, mm) between the projection of the COP on the screen and the flowing curve, as well as its velocity at a rate of 100 Hz (Zemková and Hamar, 2010). The sum of horizontal crossings of the COP trace across the flowing curve was recorded. Each test trial lasted for 30 s, with a familiarization trial carried out before the actual testing. Subsequently, three trials of ML were performed, with a 60-s rest period in between. During the rest period, subjects were free to either stand relaxed or take several slow steps (Sedliak, et al., 2013). The best of three trials was used.

##### 2.5.1.3 10 Meters test for maximal walking speed

Participants were instructed to walk as fast as they could for 10 m without any assistance (Bohannon et al., 1996; Bohannon, 1997; Wolf et al., 1999). Their walking time was measured electronically using a wireless photocell timekeeping system (Microgate, Bolzano, Italy). The maximum walking speed (MWS) in meters per second was calculated based on the fastest time from three trials. Participants were instructed to keep one foot on the ground at all times. The researchers observed the participants’ walking technique without giving any verbal encouragement during the test.

##### 2.5.1.4 The lower limbs muscular strength (maximal and relative voluntary contraction)

The strength of the lower limbs was assessed by measuring the maximum voluntary contraction (MVC) of the knee joint, using an adjustable isometric dynamometric chair (ARS dynamometry, S2P Ltd., Ljubljana, Slovenia) (Bily et al., 2016; Šarabon et al., 2020). The proctol was performed according to Sarabon et al. (2013). Each participant’s anthropometric parameters were taken into account when adapting the isometric dynamometric chair (90-degree angle in the knee joint). Prior to the testing, participants performed three warm-up trial attempts: at 50%, at 75%, and at 90% of their maximum effort. The resting period was set from 10 to 15 s between each attempt. Subsequently, each participant carried out three trials of unilateral knee isometric extension with maximal effort, with a one-minute rest interval between each trial. The participants’ dominant lower limb was determined via a health questionnaire, which was filled out and submitted before entering the study. Verbal encouragement was given to the participants throughout the test. The trial that yielded the highest force was selected for further analysis using the ARS (Analysis & Reporting Software). The relative values of maximal MVC (rel. MVC) were calculated by dividing the maximal MVC of participants by their actual body weight.

##### 2.5.1.5 Stair climb test

A stair climb test (SCT) was conducted to measure lower body strength, balance, and the ability to ascend and descend stairs (Dobson, et al., 2013; Iijima, et al., 2019; Coleman et al., 2020). Participants were instructed to ascend and descend the stairs as quickly as possible while ensuring their safety. They were advised not to overexert themselves and to use handrails if required, which were recorded. A practice trial was conducted with the researcher guarding for safety. The same set of stairs was used for both pre and post-tests. Each participant ascended and descended 11 stairs with a step height of 20 cm. The time was measured manually using a stopwatch by at least two researchers, and the average was taken as the resulting time. Timing begins on the signal to start and terminates when the participant returns with both feet to the ground level. The time was measured separately in the direction of ascent and descent, and no verbal encouragement was provided during the test.

### 2.6 Statistical analysis

All statistical analyses were carried out using SPSS for Windows (SPSS version 23.0; IBM Corp., Armonk, NY). Data are presented means and standard deviations (SD) were obtained by conventional statistical methods. To begin with, the Shapiro-Wilk test was performed for all variables to determine the normality of data distribution. Then, Levene’s test was used to check for homogeneity of variance. If the data were normally distributed, the paired-sample t-test was performed, otherwise, the Wilcoxon signed rank test was performed. The significance level was set at *p* < 0.05 (two-tailed). Repeated measures analysis of variances (ANOVA) was used to compare time and group interaction (time × group) and training intervention time effect (pre vs. post-testing) × three groups (SMT vs RET vs CG). Post hoc comparisons were performed using the Bonferroni correction. Assumptions of sphericity were evaluated using Mauchly’s test. Where sphericity was violated (*p* < 0.05), the Greenhouse-Geisser correction factor was applied. The *post hoc* statistical power of the sample size was calculated with G*Power (Version 3.1.9.6, Institut für Experimentelle Psychologie, Düsseldorf, Germany). The power for the number of subjects within the study sample was 1 − β = 0.701 with α = 0.05. To compensate for the lower sample size power, additional methods were used. Specifically, the effect size was evaluated using a partial eta squared (η^2^), with >0.01, >0.059, and >0.139 indicating a small, medium and large effect, respectively. Cohen’s d was used to detect effect size in absolute differences (pre vs post-testing) with the following interpretation: small effect (0.2 ≤ d ≤ 0.5), medium effect (0.50 ≤ d < 0.80), and large effect (d > 0.8) (Cohen, 1988).

## 3 Results

The overall results of the study are shown in Table 1. All data are presented as means and standard deviation (SD).

**TABLE 1:** Overall parameters before and after the experimental period.

Parameters	CG	RET	SMT	ANOVA 3 × 2										
	PRE	POST	*p*	ES	PRE	POST	*p*	ES	PRE	POST	*p*	ES	Time	time*group
													*p*	*p*
BW (kg)	66.99 ± 7.63	67.18 ± 7.90	.529	0.02	67.23 ± 8.82	67.33 ± 9.80	0.823	0.01	66.43 ± 9.38	65.78 ± 9.04	0.092	0.07	0.574	0.209
MM (kg)	23.16 ± 2.22	23.39 ± 2.05	0.345	0.11	23.13 ± 2.23	23.40 ± 2.85	0.346	0.11	22.53 ± 2.27	22.84 ± 2.34	0.173	0.13	0.060	0.968
FM (kg)	24.68 ± 4.71	24.55 ± 5.66	0.800	0.02	24.42 ± 6.58	24.12 ± 6.37	0.350	0.05	24.45 ± 7.00	23.23 ± 7.17	**0.002**	0.28	0.016	0.098
VMeo (mm.s^−1^)	11.48 ± 2.83	11.62 ± 2.73	0.793	0.01	15.24 ± 6.76	15.87 ± 7.44	0.400	0.01	11.98 ± 3.92	9.08 ± 1.94*	**0.028**	0.94	0.164	**0.013**
Vmec (mm.s^−1^)	16.55 ± 4.40	19.68 ± 5.66*	**0.050**	0.60	26.72 ± 14.03	25.34 ± 11.42	0.522	0.11	20.32 ± 8.15	14.38 ± 2.87*	**0.013**	0.97	0.184	**0.006**
MD (mm)	8.26 ± 1.78	8.94 ± 1.47	0.152	0.42	9.29 ± 2.14	8.45 ± 1.44	0.307	0.46	10.29 ± 1.08	6.43 ± 0.80**	**0.000**	4.06	**0.000**	**0.000**
MVCd (N.m^−1^)	112.48 ± 25.53	109.56 ± 27.08	0.476	0.11	100.86 ± 33.32	117.28 ± 35.29**	**0.004**	0.48	106.08 ± 16.93	123.65 ± 21.46**	**0.000**	0.93	**0.000**	**0.001**
MVCnon-d (N.m^−1^)	111.94 ± 24.18	110.01 ± 24.66	0.578	0.08	103.92 ± 36.09	115.24 ± 34.23*	**0.018**	0.31	100.95 ± 19.55	113.79 ± 19.67**	**0.004**	0.65	**0.001**	**0.011**
rel. MVCd (N.m^−1^)	1.70 ± 0.46	1.66 ± 0.49	0.482	0.08	1.52 ± 0.51	1.75 ± 0.48**	**0.001**	0.46	1.62 ± 0.30	1.90 ± 0.37**	**0.000**	0.83	**0.000**	**0.000**
rel. MVCnon-d (N.m^−1^)	1.69 ± 0.40	1.66 ± 0.42	0.556	0.07	1.56 ± 0.54	1.72 ± 0.49**	**0.007**	0.31	1.54 ± 0.33	1.74 ± 0.29**	**0.002**	0.64	**0.000**	**0.000**
10 m MWS (m.s^−1^)	1.95 ± 0.20	2.01 ± 0.15	0.273	0.34	1.86 ± 0.24	1.94 ± 0.24	0.397	0.33	1.80 ± 0.16	1.87 ± 0.20	**0**.100	0.39	0.062	0.978
SCTa (m.s^−1^)	0.84 ± 0.07	0.79 ± 0.11	0.099	0.54	0.79 ± 0.10	0.81 ± 0.07	0.332	0.25	0.66 ± 0.06	0.75 ± 0.08**	**0.000**	1.27	0.114	**0.001**
SCTd (m.s^−1^)	1.00 ± 0.13	0.91 ± 0.12*	0**.010**	0.71	0.86 ± 0.15	0.89 ± 0.10	0.298	0.29	0.73 ± 0.09	0.86 ± 0.11**	**0.000**	1.17	0.204	**0.000**

Data are presented as mean ± SD. CG, control group; RET, resistance-endurance training; SMT, sensorimotor training; p, *p*-value; ES, effect size; BW (kg), body weight; MM (kg), muscle mass; FM (kg), fat mass; VMeo (mm.s^−1^), velocity-mean with eyes open; VMec (mm.s^−1^), velocity-mean with eyes close; MD (mm), mean-distance; MVCd (N.m^−1^), maximal voluntary contraction of the dominant leg; MVCnon-d (N.m^−1^), maximal voluntary contraction of the non-dominant leg; rel. MVCd (N.m^−1^), relative maximal voluntary contraction of the dominant leg; MVCd (N.m^−1^), relative maximal voluntary contraction of the non-dominant leg; 10 m MWS (m.s^−1^), 10 m maximal walking speed; SCTa (m.s^−1^), Stair climb test–ascend; SCTd (m.s^−1^), Stair climb test - descend. The bold values indicate statistically significant differences.

### 3.1 Body composition

**FIGURE 4:**
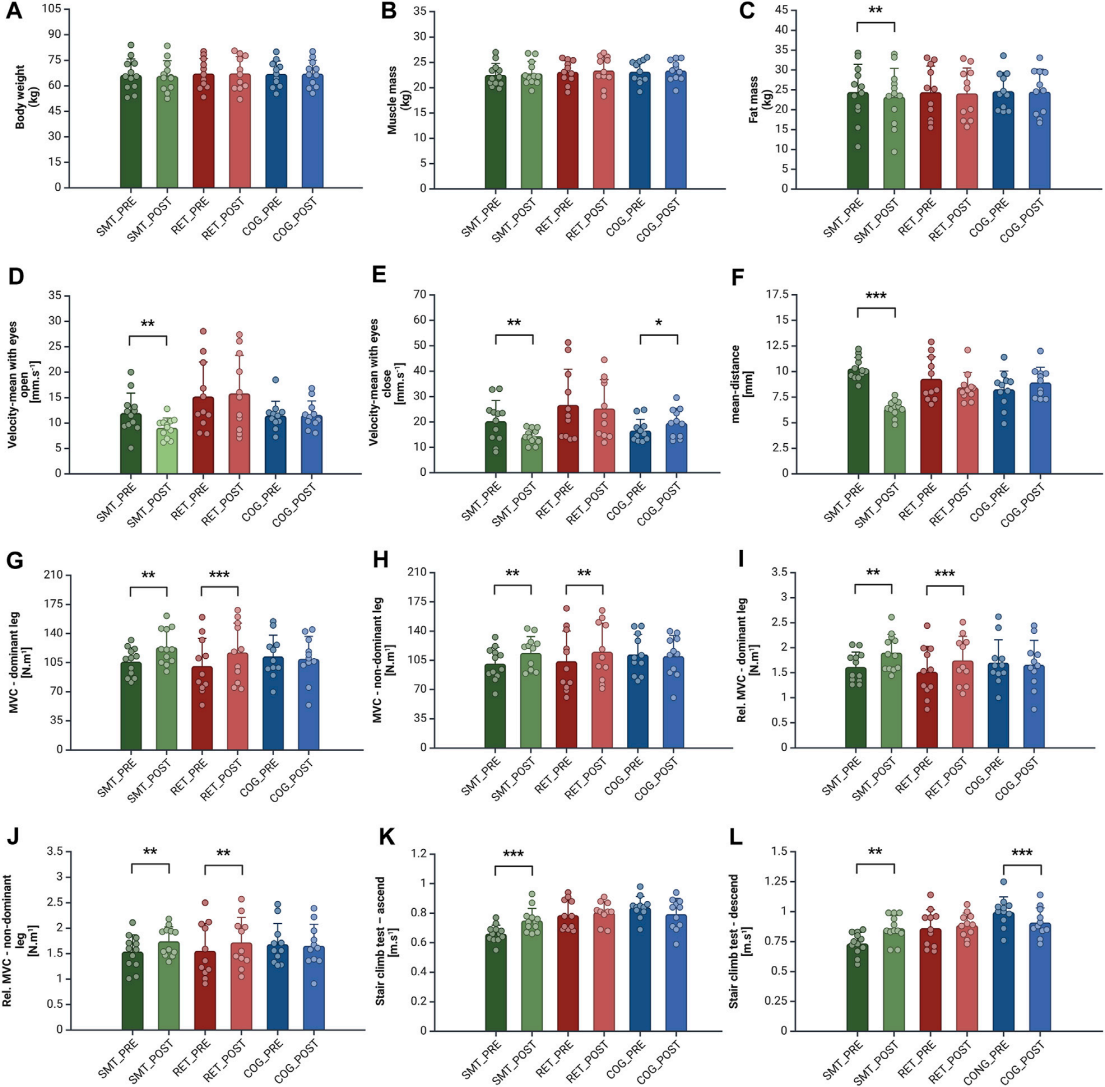
Balance and Physical Performance Differences in Measured Parameters in Each Experimental Group Before and After the Intervention. Legend: body weight **(A)**, muscle mass **(B)**, fat mass **(C)**, velocity-mean with eyes open **(D)**,velocity-mean with eyes closed **(E)**, mean-distance **(F)**, maximal voluntary contraction of the dominant leg **(G)**, maximal voluntary contraction of the non-dominant leg **(H)**, relative maximal voluntary contraction of the dominant leg **(I)**, relative maximal voluntary contraction of the non-dominant leg **(J)**, stair climb test–ascend **(K)**, stair climb test - descend **(L)**. Column bars represent the mean, error bars represent the standard deviation (SD) and dots represent individual values. *Represents statistical significance *p* < 0.050, **p < 0.010, ***p < 0.001 (Created with BioRender.com).

Body composition parameters, such as body weight (kg), muscle mass (kg), and fat mass (kg) are presented in Figures 4A–C. As the results indicate, there were no significant differences in body weight in any of the groups. Also, there were no significant differences in time × group interaction or effect of time (all *p* > 0.05). Similarly, we did not observe any significant changes in muscle mass in any of the groups after the intervention. However, according to fat mass, a significant decrease was observed in the SMT group (from 24.45 ± 7.00 kg to 23.23 ± 7.17 kg, *p* = 0.002, d = 0.28, small effect). In addition, there were significant main effects of time for FM (F_1,31_ = 6.44; *p* = 0.016; η^2^ = 0.172) (Table 1).

### 3.2 Balance skills

Balance testing results, such as tests of postural sway with eyes open and closed respectively, and visual feedback balance tests are presented in Figures 4D–F. The improvement of the dynamic balance was observed only in the SMT group. Specifically, significant changes in the SMT group were observed in the tests of VM_eo_ (from 11.98 ± 3.92 mm/s to 9.08 ± 1.94 mm/s, *p* = 0.028, d = 0.94, large effect), VM_ec_ (from 20.32 ± 8.15 mm/s to 14.38 ± 2.87 mm/s, *p* = 0.013, d = 0.97, large effect), and MD (from 10.29 ± 1.08 mm to 6.43 ± 0.80 mm, *p =* 0.000, d = 4.06, large effect), while, as may be expected, the VM_ec_ (from 16.55 ± 4.40 mm to 19.68 ± 5.66 mm, *p* = 0.050, d = 0.60, medium effect) deteriorated in the COG group. The RET group did not show significant changes in the parameters of postural control. As shown in Table 1 a significant main effect of time was observed for parameter MD (F_1,31_ = 24.37; *p* = 0.000; η^2^ = 0.440). In addition, significant differences in time × group interaction in variables VM_eo_ (F_2,31_ = 5.06; *p* = 0.013; η^2^ = 0.246), VM_ec_ (F_2,31_ = 6.15; *p* = 0.006; η^2^ = 0.28) and MD (F_2,31_ = 24.61; *p* = 0.000; η^2^ = 0.614) was present (Table 1). In this case, a significant main effect of group was also found in variables VM_eo_ (F_2,31_ = 3.97; *p* = 0.029; η^2^ = 0.204) and VM_ec_ (F_2,31_ = 3.94; *p* = 0.030; η^2^ = 0.202), following Bonferroni *post hoc* test significant differences between SMT vs RET (*p* = 0.034 and *p* = 0.044, respectively) was observed.

### 3.3 Muscle strength of lower body

The strength metrics of maximal (MVC) and relative muscle voluntary contraction (rel. MVC) are presented in Figures 4D–G. In the RET group, significant changes were observed in both MVC_d_ (+16.3%, *p =* 0.004, d = 0.48, medium effect) and MVC_non-d_ (+10.9%, *p =* 0.018, d = 0.31, small effect). Similarly, the SMT group also showed improvements in both parameters, MVC_d_ (+16.6%, *p =* 0.000, d = 0.93, large effect) and MVC_non-d_ (+12.7%, *p =* 0.004, d = 0.65, medium effect). As shown in Table 1 a significant main effect of time in the variables MVC_d_ (F_1,31_ = 22.81; *p* = 0.000; η^2^ = 0.424) and MVC_non-d_ (F_1,31_ = 13.30; *p* = 0.001; η^2^ = 0.300) was observed, as well as significant differences in time × group interaction for both variables, MVC_d_ (F_2,31_ = 9.29; *p* = 0.001; η^2^ = 0.375) and MVC_non-d_ (F_2,31_ = 5.29; *p* = 0.011; η^2^ = 0.255), respectively. A similar trend was observed in the increments of rel. MVC_d_ (+15.6%, *p =* 0.001, d = 0.46, medium effect) and rel. MVC_non-d_ (+10.3%, *p* = 007, d = 0.31, small effect) in the RET group, where both parameters improved. The SMT group also displayed enhancements in these parameters, rel. MVC_d_ (+17,3%. *p* = 0.000, d = 0.83, large effect) and rel. MVC_non-d_ (+13%, *p =* 0.002, d = 0.64, medium effect). Similarly, there were significant main effects of time in rel. MVC_d_ (F_1,31_ = 13.30; *p* = 0.000; η^2^ = 0.475) and MVC_non-d_ (F_1,31_ = 15.27; *p* = 0.000; η^2^ = 0.330). In addition, significant differences in time × group interaction in rel. MVC_d_ (F_2,31_ = 11.43; *p* = 0.000; η^2^ = 0.424) and MVC_non-d_ (F_2,31_ = 6.33; *p* = 0.000; η^2^ = 0.290) was present (Table 1).

Finally, no significant differences in the MVC and rel. MVC was observed in the COG group.

### 3.4 Physical performance

The measurements of the 10 m MWS, SCT_a_ and SCT_d_ are presented in Figures 4H, I. In all tested groups, there were not observed any significant changes in the 10 m MWS. Also, no significant effect of time or time × group interaction (all *p* > 0.05). However, as shown in Table 1, there were significant differences in time × group interaction in variables SCT_a_ (F_2,31_ = 9.26; *p* = 0.001; η^2^ = 0.374) and SCT_d_ (F_2,31_ = 14.57; *p* = 0.000; η^2^ = 0.485). Also, in this case, the main effect of the group in both variables SCT_a_ (F_2,31_ = 7.35; *p* = 0.002; η^2^ = 0.322) and SCT_d_ (F_2,31_ = 5.99; *p* = 0.006; η^2^ = 0.279) was observed. Using the Bonferroni *post hoc* test, there were significant differences between SMT vs RET (*p* = 0.019) and SMT vs CG (*p* = 0.004) in variable SCT_a_. In the second variable, SCT_d_ the Bonferroni *post hoc* test showed significant differences between SMT vs CG (*p* = 0.005).

## 4 Discussion

To quantify the effects of interventions on the parameters of static balance while standing upright with and without visual control were evaluated. The results showed that e female older adults, underwenting a 10-week program of sensorimotor exercises significantly improved the mean velocity of COP of postural sway in both upright positions, with eyes open as well as with eyes closed. These results were not particularly unexpected as they are in line with similar studies. Maćkowiak et al., (2015) showed a significant positive effect on balance in elderly women over 50 years with impaired vision after 6 weeks of sensorimotor intervention. A similar study, however in substantially younger subjects, has been carried out by Pau et al., (2012). After 6 weeks of balance training group of young volleyball players showed smaller sway areas while standing with eyes closed. The intervention also reduced anteroposterior COP displacements while standing on a non-dominant limb.

The improvement of postural stability due to sensorimotor training can be attributed to adaptive changes in the physiological mechanisms involved within its control. This complex motor ability depends primarily on multiple kinesthetic processes (Horak, 2006) providing stimuli to the CNS, which, after appropriate processing, sends efferent control commands to postural sway controlling muscles.

Theoretically, sensorimotor training may enhance the sensitivity of proprioceptors providing more precise signals from muscles, tendons, and joints enabling more efficient CNS processing and more accurate real-time regulation of postural muscle activity. Such a concept is supported by Hewett et al., (2002), who claim that sensorimotor training can improve the activation of proprioceptors, hence providing more precise information on body parts position. In this way, more sensitive proprioceptors can partially compensate for missing visual input in dark environments or in individuals with visual impairments. In addition, according to the study by Aman et al. (2015), stimulation of proprioceptors also leads to cortical reorganization, contributing to more efficient sensorimotor function.

This may be of particular benefit for older adults, in which missing visual control without efficient compensation by proprioceptive input leads to a more pronounced impairment of postural control (Maki and McIlroy, 2006). Such a lack of visual impairment compensation is one of the major factors contributing to an increased risk of falling in older adults. Therefore, improved proprioception due to specific sensorimotor training can be an important factor in the prevention of falls in the elderly population.

As mentioned earlier, sensorimotor (SM) training can also positively affect the reorganization of the sensorimotor cortex. This can be considered as a crucial mechanism for efficient learning and relearning strategies, and also for refining motor patterns that are important for the alignment of the human body to the changing requirements of the environment (Machado et al., 2010). Such a central adaptation could also, at least partially, contribute to better results in the test of visual feedback control of body position after sensorimotor intervention in the SMT group. However, pronounced improvement in this test may also be attributed to the fact that the visual feedback control of the body position was an integral part of sensorimotor training.

An increase in strength can be considered as the third mechanism of improvement of balance parameters observed in the present study. As expected, both RET and SMT groups improved in the absolute as well as the relative muscle strength in both dominant and non-dominant lower extremities without any significant differences between these two groups. These findings are in close agreement following studies of Oreská et al., (2020), Pinto et al., (2014), demonstrating comparable gains in the lower extremities’ maximal strength.

As underlying mechanisms of strength gain are traditionally considered functional changes such as enhanced neural activity at the onset of voluntary actions, which may include increases in motor unit recruitment, enhanced maximal motor unit firing rates, refinement of muscle activation patterns, elevated spinal motor neuronal excitability, increased efferent motor drive, changes in the pennation angle or the length-tension relationship of muscles, optimizing force generation as well as changes in agonist coactivation (Hakkinen and Komi, 1986; Aagaard and Andersen, 2010).

Another mechanism to consider is muscle hypertrophy. However, should this mechanism play a role, the intensity of muscle contractions must exceed the minimal intensity of 65% of 1 RM (Kraemer et al., 2002). Progression models in resistance training for healthy adults of 1 RM and training duration should exceed 6–8 weeks and even longer (9–15 weeks) in the older adults (Lam et al., 2018).

As in SMT intensity of muscle contractions necessary to meet requirements of corrective movements were of horizontal body position remained well below level necessary to stimulate morphological changes, muscle hypertrophy was unlikely to take place in subjects undergoing sensorimotor intervention. In addition, this adaptive mechanism could hardly occur because a period of intervention program (10 weeks) was in the range, where hypertrophy could be scarcely barely expected. On the other hand, slight hypertrophy could take place in the RET group, where the intensity of muscle contraction was sufficient and the duration of intervention reaching or slightly exceeding the minimum response time for morphological changes.

Strength improvement without typical resistance exercise only due to sensorimotor intervention has been observed by Granacher et al. (2010). His subjects increased their peak strength of lower extremities after balance training on unstable wobble boards as well as parameters of postural control. He attributes this improvement to real neuroregulatory adaptations rather than learning effects.

It has been shown by several studies (Hess and Woollacott, 2005; Holviala et al., 2006; Zouita et al., 2020; Amato et al., 2022) that improvements in muscle strength were concomitant with better postural sway control.

However, the results of our study did not provide clear evidence to support such a concept. Sensorimotor intervention led to a significant parallel increase in muscle strength (the maximum absolute and relative values of knee extensors on both dominant and non-dominant limbs) and parameters of balance only in SMG. Improvement of strength due to combined RE training was surprisingly not concomitated with balance parameters.

SM training, especially when done on unstable surfaces like a spring-loaded stabilographic board can improve strength and motor coordination. This is because such training increases muscle activation, improves and boosts motor unit recruitment. These neuromuscular alterations are thought to better stabilize the center of mass while standing on a stable or changing base of support (Cuǧ et al., 2016). Surprisingly similar effect has been observed after SMT intervention and in addition, there was no significant difference between both groups. This implies that in terms of strength, both types of training had practically the same effect.

An increase in muscle strength observed in the RET group plays a key role in the prevention as well as reverting of functional deterioration caused by ageing. Level of strength directly affects more complex locomotor skills such as walking, namely, at higher speeds. Its impairment is one of the crucial factors affecting older adults’ life quality and health status (Bohannon, 1997; Slobodová et al., 2022; Stanaway et al., 2011). However, our results did not support such a simple concept. Despite of increase in strength in both groups, none of them improved 10 m maximum walking speed. On the other hand, in the stair climb test only the SMT group, which improved not only in strength but also in parameters of balance, increased vertical velocity significantly. This indicates that daily living activities may be affected more by sensorimotor functions than strength capabilities itself. This is also in line with the findings of Kim and Jang (2021) showing that balance skills, both static and dynamic, engaging the visual and sensorimotor feedback from the environment have a significant impact on walking speed.

We might presume that sensorimotor function might play a more important role in more demanding daily life activities such as walking up and down the stairs than in simple level walking or parameters of postural sway. Similar findings, however, in young athletes are supported by the results of Zacharakis et al. (2020) who examined the impact of a balance and proprioceptive training program on static and dynamic balance and technical skills in youth basketball players. The experimental group of girls significantly improved in dynamic balance and basketball technical skill passing accuracy scores, but not postural static balance.

The conclusions of this study are also in line with our results showing significant improvement in both stair ascending and descending speed tests only in SMT, but not in RET group. Another factor to consider while explaining the lack of significant changes in 10 m maximal walking speed is the fact that the average maximal speed in both groups before the intervention was on the level of the extremely fit population (Bohannon, 1997; Stanaway et al., 2011). It has been shown that subjects featuring an already high level of a particular function tend to exert less pronounced responses to training stimuli than those staring at a lower level.

Subsequently, it is noteworthy to mention that even earlier mentioned daily tasks such as walking speed on flat surfaces along with ascending or descending stairs are directly determined by individuals’ balance skills, which naturally tend to deteriorate throughout the life span, as it is well scientifically proven (Vereeck et al., 2008; Soto-Varela et al., 2016; Park and Lee, 2020). Therefore, it’s in the best interest of seniors to take steps to improve this ability as much as possible or, at the very least, slow down its decline. It’s important to note that balance is a crucial component of coordination skills and is reflected in virtually all movement structures.

Balance, whether static or dynamic, is the decisive neuromotor ability of humans for everyday activities (Gambetta and Gray, 1995). Static balance refers to the ability to maintain an upright position and other static postures as squatting or sitting under stable conditions.

Dynamic balance is crucial for maintaining balance under unstable conditions, typically during activities such for example, walking, running, skiing, cycling, or climbing stairs. It also affects the quality of performing technically demanding skills in sports like for example, basketball, volleyball, football, or gymnastics (Pau et al., 2012; Zacharakis et al., 2020; Kenville et al., 2021).

Both static and namely, dynamic balance also play important roles in compensation as the sudden distortion of body position, a process of crucial importance in the prevention of falls and resulting injuries. This is especially important in the elderly population.

Our study proves that SM training is highly effective for the elderly population improving their postural stability and visual feedback control of regulation of horizontal corrective body movements following the curve displayed on the monitor.

Results of the study have also revealed, that sensorimotor training increased the maximal and relative strength of the lower extremities and the vertical speed of walking up and down stairs. As seniors age, they may avoid conventional strength training due to cardiovascular problems, injuries, or physical limitations. However, strength and balance decline put them at risk of falls. In terms of strength development, SM training is a promising alternative to improve overall strength and balance, reducing the risk of falls and maintaining independence in the older adults with no previous experience of training.

### 4.1 Strengths and limitations

Besides the original approach of the current study, there might be several notable limitations. It could be argued that the study had a small number of subjects in each experimental group. The results also may not be directly translated to the overall population of the elderly as only older females were measured. Thus, the inclusion of the male population then may give us a broader picture, of how this sensorimotor training affects the elderly population in general. Additionally, DXA body composition measurement would provide us with more detailed information about the effect of hypertrophy or neuromuscular adaptation on muscle strength, caused by training intervention. Moreover, deeper testing of neuromuscular structural and functional changes, like EMG testing before and after the intervention or each training session, could reveal quicker activation of muscle fibers in the muscles responsible for the balance. Another limitation in explaining the results could be the absence of muscle biopsies, which could offer a more comprehensive view of the morphological changes at the muscle level. The habitual physical activity of the participants was not assessed in this study. The beneficial influence of physical activity in general for the prevention of age-related sarcopenia was suggested in many studies (Steffl et al., 2017). Lastly, it could be hypothesized that in the SMT group, the post-testing results in the specific test of copying the curve might be affected by using a slightly similar stimulus, which was used within the intervention.

On the positive side, the study utilized an innovative system of unstable platforms as a unique training method for the elderly population. Another benefit to consider is the impact on their balance and strength abilities, which are crucial for their daily tasks, as supported by the aforementioned results.

## 5 Conclusion

In conclusion, sensorimotor training has been proven to be highly effective in improving postural control and muscular strength in older adult individuals, comparable to resistance-endurance training. By targeting sensory integration, motor coordination, and balance control, this form of training helps enhance overall stability, reduce the risk of falls, and improve functional independence in older adults. The study results indicate that sensorimotor training may serve as a suitable alternative for individuals with various health disorders that are in contradiction with classical strength and endurance training. Additionally, sensorimotor training can be beneficial for those who do not prefer or are unable to incorporate this type of exercise into their daily lives.

## Data Availability

The raw data supporting the conclusions of this article will be made available by the authors, without undue reservation.
